# A randomized controlled trial of different methods of alcohol screening and brief intervention in routine accident and emergency department care: 12-month outcomes

**DOI:** 10.1186/1940-0640-7-S1-A80

**Published:** 2012-10-09

**Authors:** Paolo Deluca, Colin Drummond, Simon Coulton, Eileen Kaner, Dorothy Newbury-Birch, Tom Phillips, Katherine Perryman, Nick Heather, Christine Godfrey

**Affiliations:** 1 Institute of Psychiatry, King's College London, London, UK; 2 National Addiction Center, Institute of Psychiatry, King's College London, London, UK; 3 Center for Health Service Studies, University of Kent, Canterbury, UK; 4 Institute of Health and Society, Newcastle University, Newcastle upon Tyne, UK; 5 Department of Health Sciences, University of York, Heslington, York, UK; 6 Humber National Health Service Foundation Trust, Willerby, UK; 7 School of Medicine, University of Manchester, Manchester, UK; 8 Division of Psychology (Emeritus), Northumbria University, Newcastle upon Tyne, UK

## 

There is a wealth of evidence on the detrimental impact of excessive alcohol consumption on physical, psychological, and social health. There also exists a substantial evidence base for the efficacy of alcohol brief intervention (BI) aimed at reducing consumption across a range of settings. Research conducted in emergency departments (EDs) has reinforced the current evidence regarding the potential effectiveness and cost-effectiveness of BI. However, the majority of this research has been conducted in a single center, and there is little evidence of the generalizability of SBI implementation across EDs. This pragmatic cluster randomized controlled trial randomized nine EDs to a combination of screening tools (the Modified Single Alcohol Screening Question [M-SASQ], the Fast Alcohol Screening Test [FAST], or the Screening and Intervention Program for Sensible Drinking modified Paddington Alcohol Test [SIPS-PAT]) and interventions (patient intervention leaflet [PIL], brief advice [BA], or brief lifestyle counseling [BLC]). The primary hypothesis was that BLC delivered by an alcohol health worker would be more effective than BA or PIL delivered by ED staff. Outcomes were assessed at six and 12 months. Overall, 5992 patients were screened for eligibility in 9 EDs; of these, 3737 (62%) were found eligible, and 1491 screened positive for an alcohol use disorder (40%). Of those who screened positive, 1204 (81%) consented to participate in the trial. The mean age of participants was 35 years, and the mean AUDIT score at baseline was 12.4. The majority of the sample was male (65%) and white (88%). At 12 months, 803 (67%) of participants were followed up. No significant differences in follow-up rates were observed between intervention groups. Overall, the proportion of participants positive for an alcohol use disorder reduced significantly by 16.3%. This reflected a significant decrease of 18.8% in the PIL group and 15.1% in both the BLC and BA groups. An adjusted logistic regression model found no significant effects of intervention group, screening approach, or baseline AUDIT score.

